# Extraction, Profiling, and Characterization of Phytosterols and Triterpenoids from Pili (*Canarium ovatum* Engl.) Pulp Oil Exhibiting Antioxidant and Antibacterial Properties

**DOI:** 10.1155/2022/6604984

**Published:** 2022-12-27

**Authors:** Nico G. Dumandan, Annie Cita T. Kagaoan, Ranelle D. P. Acda, Caren R. Tumambing, Laura J. Pham

**Affiliations:** ^1^National Institute of Molecular Biology and Biotechnology (BIOTECH), University of the Philippines Los Baños College, Los Baños 4031, Laguna, Philippines; ^2^Biozym Systems Enterprises, Bay 4033, Laguna, Philippines

## Abstract

Pili (*Canarium ovatum* Engl.), an indigenous tree found in the Philippines, is highly regarded for its fruit due to its high economic value. During processing, the pulp is often discarded as waste but contains considerable amounts of oil and bioactive minor lipid components. The present study explored the antioxidant and antibacterial properties of saponified diethyl ether extract of pili pulp oil and related this activity to the nature of compounds present in the extract through GCMS. The extract indicated the elution of 18 major compounds which are mostly cyclic triterpenic (*α*-and *β*-amyrin, lupenone, and *β*-amyrone) and phytosterol (*β*-sitosterol, brassicasterol, and stigmasterol) class of compounds. Characterization of the bioactivity of the extract showed high antioxidant activities measured by DPPH radical scavenging (EC_50_: 74.45 ± 1.29 *μ*g/mL) and lipid peroxidation inhibition (EC_50_: 3.02 ± 0.06 *μ*g/mL) activities that were comparable with that of *α*-tocopherol. Moreover, an observed bactericidal activity was demonstrated by the extract against *E. coli* and *S. typhi* with MIC values of 40 and 35 *μ*g/mL, respectively. The observed bioactivity of the pili pulp oil extract can be attributed to these compounds which may provide desirable health benefits.

## 1. Introduction

*Canarium ovatum* Engl., locally known as pili, is an indigenous tree commonly found in the Philippines which is cultivated for its edible fruit [1]. Pili nut kernel is the most valuable part of the fruit due to its high economic value owing to its increasing competitiveness in the global confectioneries market [2]. In pili nut processing, its pulp is often discarded as waste, but it contains an appreciable amount of oil and important minor lipid species such as carotenoids, phytosterols, and tocopherols [3, 4]. These nutritionally beneficial minor lipid compounds have gained considerable interest, particularly in their bioactivity which plays an important role in the development of high-value products.

Antioxidants and antimicrobial agents play a significant role in the food sector primarily because bacterial growth and lipid oxidation are the main factors that determine food quality loss and shelf-life reduction. Oftentimes, synthetic additives such as BHA/BHT are commonly added to food products to inhibit the process of lipid oxidation and microbial growth and to extend their shelf-life. However, a shift to naturally derived compounds is seen and increasingly being sought by many companies due to possible adverse effects associated with long-term intake of synthetic compounds [5, 6]. Phytosterols, including other cyclic triterpenes which constitute the majority of unsaponifiable fractions of seeds oils, are known to have several bioactive properties linked to various implications on human health, including anti-inflammatory, antioxidative, antimicrobial, cholesterol-lowering, and anticarcinogenic activities [7–12]. On the other hand, triterpenoid extracts that are rich in lupeol, betulinic acid, and amyrin have been shown to inhibit the growth of foodborne pathogenic bacteria particularly the methicillin-resistant*S. aureus*, *E. faecalis,* and *P. aeruginosa* are studied by Amoussa et al. [13] and Nzogong et al. [10]. Phenolic compounds have been shown to fuse with extracellular soluble proteins of the microbial cell wall resulting in the suppression of microbial growth and/or oxidative damage [14].

Hence, the present study explored the potential of minor lipid components in pili pulp oil as a source of phytosterols and cyclic triterpenoids with antioxidant and antibacterial properties. The interest in these naturally derived compounds is not only due to their biological activity but also to maximize the economic potential of pili pulp oil.

## 2. Materials and Methods

### 2.1. Materials

Pili (*Canarium ovatum* Engl.) fruits were obtained from a local market at Goa, Camarines Sur, Philippines. Microbial strains (*E. coli, P. aeruginosa, S. tyhpi, S. aureus*, and *B. cereus*) used for the antibacterial activity assay were obtained from the Philippine National Collection of Microorganisms (PNCM), BIOTECH, UPLB, College, Los Baños, Laguna, Philippines. All other chemicals and standards were purchased commercially.

### 2.2. Sample Preparation and Oil Extraction

Pili fruits were manually depulped by blanching in lukewarm water for about 15–20 min. The pulps were then collected and dried in a convection-type oven at 70°C overnight or until moisture content reached about less than 3%. Extraction of oil was carried out by using *n*-hexane at 1 : 4 ratio of dried pulp weight (g) to solvent volume (mL). After 12 h of extraction at room temperature with constant agitation, the oil was recovered by solvent evaporation by using a rotary evaporator.

### 2.3. Saponification and Fractionation

The unsaponifiable fraction of oil was obtained by saponification following the method of Almeida et al. [15] with some modifications. A 0.3 g of the oil sample was saponified by using 10 mL 3% w/v ethanolic potassium hydroxide at 50°C for 3 h. Then, the solution was cooled by adding 10 mL of distilled water. Subsequent fractionation of the phytosterol and triterpenoids was carried out by repeated liquid-liquid extraction by using 10 mL diethyl ether three times. The organic layers were then combined, washed twice with 10 mL of distilled water, and dried over anhydrous sodium sulfate. The saponified diethyl ether extract (SDEE) of pili pulp oil was then collected upon filtration and solvent evaporation under a stream of nitrogen gas.

### 2.4. GCMS Profiling

Profiling of SDEE was performed on a Shimadzu GCMS-QP2020 equipped with a Shimadzu AOC-20i Plus auto-injector (Shimadzu Corp., Kyoto, Japan) under electron impact ionization at 70 eV. Separation of components was carried out in SH-Rxi-5Sil MS capillary column with dimensions of 30 m × 0.25 mm ID × 0.25 *μ*m film thickness (Shimadzu Corp., Kyoto, Japan). The initial oven temperature was held at 190°C for 1 min then raised to 300°C at 15°C/min and kept constant for 10 min. Helium was used as the carrier gas with a constant flow rate of 1 mL/min. The injector, MS ion source, and MS interface temperatures were set at 310, 230, and 280°C, respectively. A sample injection of 1 *μ*L was performed in a split mode of 10 : 1 and peaks were detected in full scan acquisition mode from *m*/*z* 50 to 500. Identification of the individual components was performed by NIST mass spectral library on the basis of the mass fragment and *e*/*z* values of each component. The relative concentration of each peak was computed based on the total ion count.

### 2.5. DPPH Radical Scavenging Activity

The scavenging activity of SDEE against DPPH radical was evaluated by using the method of [16] with some modifications. Briefly, 3.8 mL of 0.07 mM DPPH solution in chloroform was mixed with 0.2 mL of the sample with varying concentrations. The mixture was incubated in the dark at room temperature for 30 min. Chloroform and *α*-tocopherol were used as a blank and positive control, respectively. The absorbance of the resulting mixture was measured at 516 nm and the percent radical scavenging activity was calculated based on the following equation:

(1) $$\begin{array}{c} DPPH\;Radi\;cal\;Scavenging\;Activity=\left[1-\frac{A_{sample}}{A_{blank}}\right]x\;100\text{.} \end{array}$$

The EC_50_ value is defined as the concentration of sample required to scavenge 50% of DPPH radical under the assayed conditions.

### 2.6. Lipid Peroxidation Inhibition Activity

A mixture of 0.1 mL of 0.1–10 *μ*g/mL of SDEE (in chloroform) and 0.5 mL of 10% egg yolk homogenate (w/v in distilled water) was mixed in a screwcapped tube and the volume was made up to 1.0 mL by adding distilled water. Then, 0.05 mL 0.07 M FeSO_4_ (in distilled water) was added, and the mixture was incubated at 37°C for 30 min to induce lipid peroxidation. A 0.05 mL of 20% (w/v) trichloroacetic acid solution (TCA) in distilled water and 1.0 mL 0.1% (w/v) thiobarbituric acid solution (TBA) in distilled water was added to the mixture, vortexed, and heated in a water bath at 80°C for 30 min. After cooling, 3 mL of butanol was added, and the mixture was centrifuged at 3000 rpm for 5 min. The absorbance of the upper layer was measured against 3 mL butanol at 512 nm. Chloroform and *α*-tocopherol were used as blank and positive controls, respectively. The lipid peroxidation inhibition activity was calculated by using the following equation:

(2) $$\begin{array}{c} Lipid\;peroxida\;tion\;inhibition\%=\left[1-\left(\frac{A_{512\;sample}}{A_{512\;blank}}\right)\right]x100\text{.} \end{array}$$

The EC_50_ value is defined as the concentration of sample required to inhibit 50% of lipid peroxidation under the assayed conditions.

### 2.7. Antibacterial Activity

#### 2.7.1. Disk Diffusion Assay

Qualitative antimicrobial activity of SDEE against *S. aureus, B. cereus, E. coli, S. typhi,* and *P. aeruginosa* was carried out by disk diffusion method [17]. Briefly, a suspension of the test microorganism standardized to 0.5 McFarland with approximately 1.5 × 10^8^ CFU/mL was uniformly spread onto individual solid media plates of Muller–Hinton agar by using a sterile cotton swab. Disks of 6 mm in diameter were impregnated, until saturation, with 0.1 mg/mL of the extract. The disks were then allowed to dry placing in the inoculated agar. Chloramphenicol and chloroform served as positive and negative controls, respectively. The inoculated plates were incubated at 37°C for 18–24 h. Antimicrobial activity was evaluated by measuring the zone of inhibition against the test organisms.

#### 2.7.2. Minimum Inhibitory Concentration Assay

The MIC of the extract exhibiting sensitivity to the tested microorganism based on the disk diffusion assay was determined following the methods outlined by Eloff [18] with some modifications. First, a stock solution of the extract (1 mg/mL) was prepared in Meuller Hinton broth supplemented with 0.02% Tween 80. The solution was then sonicated for 30 s and vortex homogenized for 2 min to obtain a stable emulsion. Serial dilutions of the extract in broth were prepared in microtubes of 1 mL with a concentration range from 0.1 to 100 *μ*g/mL. Then, 295 *μ*L of each dilution was transferred into a 96-well microplate. A 5 *μ*L of test bacterial suspension (1.0 × 10^6^ CFU/mL) was inoculated to obtain a final concentration of 1.67 × 10^4^ CFU/mL and a final volume of 300 *μ*L per well. The inoculum (positive control) and culture medium (negative control) were put into the first column of the microplate, and the chloramphenicol antibiotic control ranging from 0.5 to 75 *μ*g/mL was in the final column. Each plate was wrapped loosely with cling film to prevent dehydration during incubation for 18–24 h at 37 °C. Subsequently, 10 *μ*L of bacterial growth indicator, resazurin at 6.75 mg/mL, was added to wells, which were then incubated for 30 min at 37 °C. The lowest concentration of the extract that visually showed no growth was determined as MIC.

### 2.8. Statistical Analysis

All experiments and measurements were carried out in triplicate. The data were presented as mean ± SEM and were analyzed by using one-way ANOVA. Means were compared using Tukey's post hoc comparison test by using R software (version 4.2.1, R Foundation for Statistical Computing, Vienna, Austria). Significant differences between means were determined at *P* < 0.05.

## 3. Results and Discussion

### 3.1. Oil Extraction, Fractionation, and GCMS Profiling of the Diethyl Ether Unsaponifiable Extract of Pili Pulp Oil

**Figure 1:**
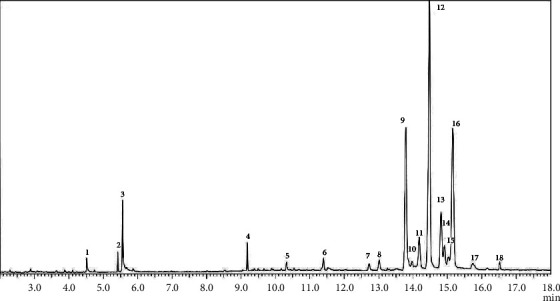
GCMS chromatogram of the saponified diethyl ether extract of pili pulp oil. Refer to Table 1 for peak identities.

Extraction of oil revealed 23.92 ± 0.21% yield, expressed in dry weight basis of pulp. The unsaponifiable fraction of oil obtained from diethyl ether extraction showed 1.0294 ± 0.0501%. These values were slightly lower than our previous findings [3] which can be attributed to the varietal differences of pili fruit used, for which this was also observed in the report of Tugay et al. [4] with 0.47 to 0.53% UM content depending on the fruit's variety. Analysis of the chromatogram obtained from the SDEE extract indicated the elution of 18 major compounds which are mostly triterpenic and phytosterol class of compounds (Figure 1, Table 1).

**Figure 2:**
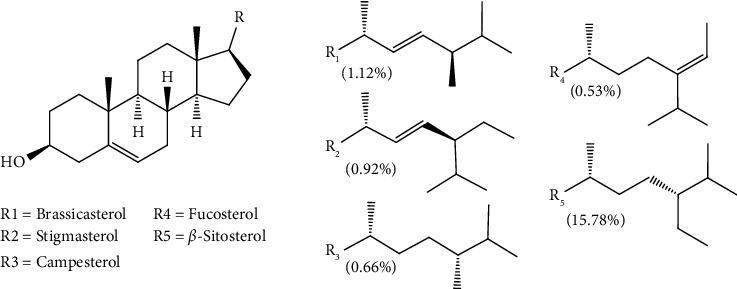
Structures of phytosterols identified in the saponified diethyl ether extract of pili pulp oil. Values in parenthesis denote relative abundance in percent of total ion count.

**Figure 3:**
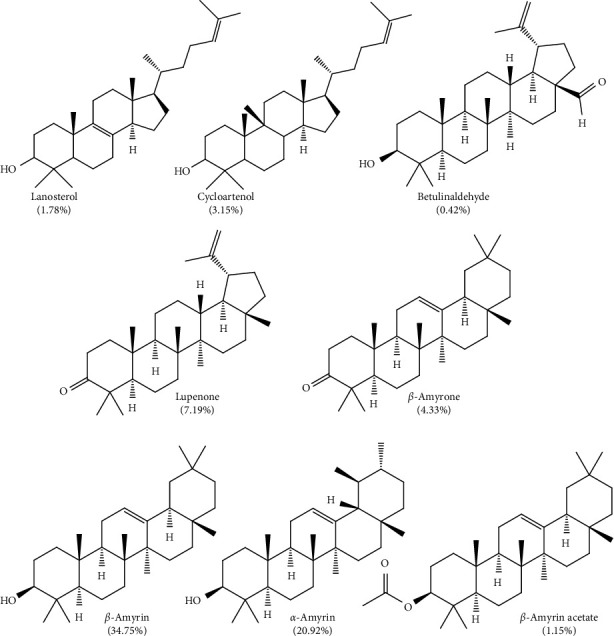
Structures of cyclic triterpenoids identified in the saponified diethyl ether extract of pili pulp oil. Values in parenthesis denote relative abundance in percent of total ion count.

In terms of relative abundance with respect to total ion count (TIC), *β*-Amyrin showed the highest abundance of around 34.75%, followed by *α*-amyrin and *β*-sitosterol with 20.92 and 15.78%, respectively. Other phytosterols identified in SDEE include brassicasterol, stigmasterol, campesterol, and fucosterol (Figure 2). These were consistent with our previous study where the major sterols present are stigmasterol and campesterol [3]. In addition, it also contains other pentacyclic triterpenes such as lupenone, amyrone, cycloartenol, and betulinaldehyde (Figure 3).

Amyrin, both *α*-and *β*-isomers, are generally found in different plant part extracts such as in *C. tramdenum* bark which contains 0.03 mg/g *β*-amyrin [19]. Areal parts of *M. barteri* and *S. longifolia* also contain high amounts of *α*-amyrin as reported by Ce et al. [20] and Saeidnia et al. [21]. Other than plant parts, amyrin along with other sterols were also detected in oil samples such as in olive, flaxseed, and camellia seed oils [22, 23].

### 3.2. Antioxidant Activity

The antioxidant activity of the SDEE was analyzed in terms of scavenging activity against DPPH radical and the ability to inhibit lipid peroxidation in the egg yolk system. As shown in Table 2, SDEE showed comparable EC_50_ values of 3.02 *μ*g/mL with that of *α*-tocopherol (2.92 *μ*g/mL), a known antioxidant compound, though a moderate activity was observed in terms of its DPPH radical scavenging activity with a slightly higher EC_50_ value of 74.45 *μ*g/mL in SDEE as compared to 65.91 *μ*g/mL in control.

Terpenoids and sterols, in general, have been shown to exert antioxidant properties owing to their hydroxyl group that participates via hydrogen atom transfer or single electron transfer mechanism in quenching reactive oxygen species [24, 25]. It has been demonstrated that phytosterol components such as campesterol, *β*-sitosterol, and stigmasterol have been shown to exhibit scavenging activities against DPPH radical and provide hepato- and neuro-protection of hydrogen peroxide-induced oxidative stress levels [25, 26]. Isomers of amyrin isolated from *Myrcianthes pungens* leaves revealed high antioxidant activity with antioxidant protection equivalent to the Trolox of 137 and 129% for *α*-and *β*-amyrin, respectively, at 500 *μ*g/mL [27].

### 3.3. Antibacterial Activity

The antibacterial potential of SDEE against 5 common food pathogens is shown in Table 3. The extract was found to exhibit significant inhibitory activity against *E. coli* and *S. typhi* with 10.3 and 11.7 mm zone of inhibition, respectively, at 0.1 mg/mL. The MIC showed that 40 and 35 *μ*g/mL of the extract were able to inhibit the growth of *E. coli* and *S. typhi*, respectively. The extract was not active or did not show antibacterial activity in *S. aureus*, *B. cereus*, and *P. aeruginosa* at 0.1 mg/mL.

The antibacterial activity of SDEE can be attributed to the high amyrin content which was also observed by Abdel-Raouf et al. [28] in several algal extracts containing *β*-amyrin exerting antibacterial activities against the 5 pathogens tested in this study. In a separate study, hexane extract of *Manilkara subsericea* fruit which is mostly *α*-and*β*-amyrin acetate showed a MIC value of 250 *μ*g/mL against *S. aureus* [29]. Aside from amyrin, Alawode et al. [30] reported that phytosterols, specifically stigmasterol and *β*-sitosterol that were isolated from *Icacina trichantha*, showed high antimicrobial properties against *B. subtilis*, *E. coli,* and *C. albicans*.

The regulatory activity of amyrin including other triterpenoids in disrupting pathways responsible for cell division and protein synthesis, as well as destabilization of bacterial cell membrane and inhibition of cell growth are some of the plausible mechanisms of action of these compounds [31]. Furthermore, these compounds also cause disorganizing effects on cardiolipin-rich domains present in the membrane of *E. coli* as demonstrated by Broniatowski et al. [32].

## 4. Conclusion

Extraction, saponification, and diethyl ether fractionation of the unsaponifiable matter of pili pulp oil showed strong antioxidant properties as measured by its scavenging activity against DPPH radical and lipid peroxidation inhibition activities and are found to be comparable to *α*-tocopherol, a known antioxidant compound. The extract also exerts antibacterial activities against *E. coli* and *S. typhi* which are bacterial pathogens of concern, especially in food preparation. GCMS profiling revealed 18 compounds majority of which are cyclic triterpenes and phytosterols such as *α*- and *β*-amyrin, lupenone, *β*-sitosterol, brassicasterol, and stigmasterol. Our results suggest that these bioactive compounds are responsible for the observed bioactivities which may provide desirable health benefits.

## Figures and Tables

**Table 1:** GCMS profiling of the saponified diethyl ether extract from pili pulp oil.

Peak no	Name of the compound	*R*_*T*_ (min)	Molecular formula	Molecular weight	Relative abundance (% of TIC)
Phytosterols and triterpenoids					
4	Squalene	9.181	C_30_H_50_	410.7	1.37
5	Stigmasta-3,5-diene	10.335	C_29_H_48_	396.7	0.42
6	Brassicasterol	11.400	C_28_H_46_O	398.7	1.12
7	Campesterol	12.719	C_28_H_48_O	400.7	0.66
8	Stigmasterol	13.007	C_29_H_48_O	412.7	0.92
9	*β*-Sitosterol	13.781	C_29_H_50_O	414.7	15.78
10	Fucosterol	13.961	C_29_H_48_O	412.7	0.53
11	*β*-Amyrone	14.188	C_30_H_48_O	427.7	4.33
12	*β*-Amyrin	14.483	C_30_H_50_O	426.7	34.75
13	Lupenone	14.816	C_30_H_48_O	424.7	7.19
14	Cycloartenol	14.912	C_30_H_50_O	426.7	3.15
15	Lanosterol	15.030	C_30_H_50_O	426.7	1.78
16	*α*-Amyrin	15.159	C_30_H_50_O	426.7	20.92
17	*β*-Amyrin acetate	15.735	C_32_H_52_O_2_	468.8	1.15
18	Betulinaldehyde	16.515	C_30_H_48_O_2_	440.7	0.42

Nonterpenoid compounds					
1	Palmitic acid	4.516	C_16_H_32_O_2_	256.4	0.78
2	Phytol	5.412	C_20_H_40_O	296.5	0.86
3	Oleic acid	5.554	C_18_H_34_O_2_	282.5	3.85

**Table 2:** Antioxidant activities of the saponified diethyl ether extract pili pulp oil.

Antioxidant assay	Activity EC_50_ (*μ*g/mL)	
	SDEE	*α*-tocopherol (control)
DPPH radical scavenging	74.45 ± 1.29^a^	65.91 ± 0.05^b^
Lipid peroxidation inhibition	3.02 ± 0.06	2.92 ± 0.19

^a-b^Means ± SEM (*n* = 3) in a row without a common superscript letter differ (*P* < 0.05) as analyzed by one-way ANOVA and the TUKEY HSD test SDEE–saponified diethyl ether extract of pili pulp oil.

**Table 3:** Antibacterial activity of the saponified diethyl ether extract pili pulp oil.

Pathogen tested	Antibacterial activity			
	SDEE		Chloramphenicol (control)	
	ZOI (mm)	MIC (*μ*g/mL)	ZOI (mm)	MIC (*μ*g/mL)
*Escherichia coli*	10.3 ± 0.20	40.0 ± 0.50	15.3 ± 0.00	2.25 ± 0.00
*Salmonella typhi*	11.7 ± 0.10	35.0 ± 0.50	15.7 ± 0.10	2.55 ± 0.00
*Staphylococcus aureus*	N.A.		15.3 ± 0.10	2.25 ± 0.00
*Bacillus cereus*	N.A.		20.3 ± 0.10	15.25 ± 0.50
*Pseudomonas aeruginosa*	N.A.		11.3 ± 0.00	2.00 ± 0.50

ZOI–zone of inhibition MIC–minimum inhibitory concentration N.A. – not active (no zone of inhibition observed) SDEE–saponified diethyl ether extract of pili pulp oil.

## Data Availability

Datasets related to this article will be made available upon request.
